# Association of Polygenic Risk for Attention-Deficit/Hyperactivity Disorder With Co-occurring Traits and Disorders

**DOI:** 10.1016/j.bpsc.2017.11.013

**Published:** 2018-07

**Authors:** Ebba Du Rietz, Jonathan Coleman, Kylie Glanville, Shing Wan Choi, Paul F. O’Reilly, Jonna Kuntsi

**Affiliations:** Medical Research Council Social, Genetic and Developmental Psychiatry Centre, Institute of Psychiatry, Psychology and Neuroscience, King’s College London, De Crespigny, United Kingdom

**Keywords:** ADHD, Comorbidity, Co-occurring disorders, Genetics, Pleiotropy, Polygenic risk

## Abstract

**Background:**

A recent large-scale mega genome-wide association study identified, for the first time, genetic variants at 12 loci significantly associated with attention-deficit/hyperactivity disorder (ADHD). In this study we use a powerful polygenic approach, with polygenic scores derived from the genome-wide association study, to investigate the etiological overlap between ADHD and frequently co-occurring traits and disorders.

**Methods:**

Polygenic risk scores for ADHD derived from the mega genome-wide association study (20,183 cases and 35,191 control subjects) were computed in a large-scale adult population sample (*N* = 135,726) recruited by the UK Biobank. Regression analyses were conducted to investigate whether polygenic risk for ADHD is associated with related traits and disorders in this population sample. The effects of sex were investigated via inclusion of an interaction term in the models.

**Results:**

Polygenic risk for ADHD significantly and positively predicted body mass index (*R*^2^ = .45%; *p* = 5 × 10^−129^), neuroticism (*R*^2^ = .09%; *p* = 2 × 10^−24^), depression (*R*^2^ = .11%; *p* = 2 × 10^−13^), anxiety (*R*^2^ = .06%; *p* = 3 × 10^−4^), risk taking (*R*^2^ = .12%; *p* = 9 × 10^−25^), alcohol intake (*R*^2^ = .09%; *p* = 8 × 10^−29^), smoking (*R*^2^ = .33%; *p* = 4 × 10^−21^), alcohol dependency (*R*^2^ = .21%; *p* = 5 × 10^−6^), and negatively predicted verbal-numerical reasoning (*R*^2^ = .38%; *p* = 5 × 10^−36^). Polygenic risk scores did not significantly predict schizophrenia or bipolar disorder, although this may be because of the small number of diagnostic cases. We found no interaction effects between polygenic risk for ADHD and sex on any phenotypes.

**Conclusions:**

Our findings suggest that common genetic variation underlying risk for clinically diagnosed ADHD also contributes to higher body mass index, neuroticism, anxiety and depressive disorders, alcohol and nicotine use, risk taking, and lower general cognitive ability in the general population. These findings suggest that the co-occurrence of several traits with ADHD is partly explained by the same common genetic variants.

SEE COMMENTARY ON PAGE 577

A recent mega genome-wide association study (GWAS) was the first to identify 12 loci significantly associated with attention-deficit/hyperactivity disorder (ADHD) (1). The statistical power of this GWAS allows the investigation of aspects of the genetic etiology of ADHD and its co-occurring features through polygenic approaches. Typically in polygenic risk analyses, composite scores, known as polygenic risk scores (PRSs), are created for individuals based on the sum of their risk alleles across the genome, weighted by GWAS-derived effect sizes. These PRSs optimize the genetic signal underlying complex traits and disorders and have been widely used to investigate shared genetic etiology between phenotypes 2, 3, 4.

Previous GWAS and candidate studies failed to identify rare and common genetic variants underlying ADHD that explain more than a small fraction of its heritability 5, 6, 7, despite the high heritability of ADHD estimated at 0.76 from twin studies (8) and estimated at 0.22 to 0.32 based on single nucleotide polymorphisms (SNPs) 1, 9. The difficulty in identifying genetic variants has likely been because of low statistical power and the polygenic nature of ADHD, i.e., that risk is a consequence of many small genetic effects. This has been supported by recent polygenic studies that show that significant associations emerge when a high number of genetic variants are considered en masse 1, 10.

ADHD has a prevalence rate of around 5.3% in childhood and 2.5% to 2.9% in adulthood 11, 12, 13. While the diagnosis of ADHD is based on inattentive and hyperactive-impulsive symptoms, affected individuals often also experience other adverse conditions. Individuals with ADHD are more likely than the general population to present with higher body mass index (BMI) 14, 15, neurotic (16) and risk-taking 17, 18, 19 behavior, lower IQ scores, and conditions such as bipolar disorder (BD), depression, anxiety 20, 21, 22, 23, 24, schizophrenia 24, 25, and substance abuse 20, 21, 23, 26, 27.

Family and twin studies suggest that several of these associations between ADHD and co-occurring traits and disorders are moderately to substantially explained by genetic influences 24, 25, 28, 29, 30, 31, 32, 33, 34. Until recently, the genetic overlap between ADHD and associated traits and disorders had not been studied using genome-wide approaches; however, limited recent and yet unpublished studies using linkage disequilibrium score regression (LDSR) report significant genetic correlations between ADHD and BMI (*r*_g_ = 0.21–0.26), educational and cognitive measures (*r*_g_ = −0.25 to 0.54), depression (*r*_g_ = 0.48), BD (*r*_g_ = 0.25), schizophrenia (*r*_g_ = 0.22), and smoking (*r*_g_ = 0.38–0.48), but not neuroticism and obsessive-compulsive disorder 1, 35. No genome-wide studies have yet investigated the genetic association between ADHD and risk taking, or alcohol and drug use.

While associations between ADHD and co-occurring impairments are well documented, our knowledge of the shared etiological influences underlying these co-occurrences is still limited with regard to the magnitude and type of genetic variants implicated in the genetic associations. The advantage of using a polygenic approach to study the genetic associations between phenotypes is that 1) we use molecular genetic data that do not rely on assumptions of relatedness, as in twin studies; 2) the design captures the polygenic nature of complex traits and disorders; and this design in turn 3) increases power to detect significant effects in studies compared with those considering only the most associated variants or candidate genes. In contrast to LDSR, the polygenic scoring method uses individual-level SNP, resulting in greater statistical power data and allowing for direct testing of interaction effects.

In this study, we use a powerful polygenic approach exploiting PRSs derived from the recently published mega GWAS on ADHD to test whether genetic variants that contribute to ADHD also influence frequently co-occurring traits and disorders in a large-scale adult population sample. A greater understanding of why ADHD often co-occurs with other impairing conditions may in turn improve preventative strategies and treatment for affected individuals. We further investigate whether the genetic overlap between ADHD and co-occurring features varies as a function of sex. Although a recent study suggested a near complete overlap of common genetic variants associated with ADHD between males and females (36), there may be sex differences in the genetic overlap between ADHD and comorbid features.

## Methods and Materials

### Discovery Sample

We used the recently published mega GWAS on ADHD as the discovery dataset (1). Summary results were downloaded from the PGC website (https://www.med.unc.edu/pgc/results-and-downloads). This GWAS contains data from 55,374 children and adults (20,183 ADHD cases and 35,191 control subjects), and 8,047,421 SNPs. Twelve independent loci were significantly associated with ADHD, and polygenic risk calculated from the GWAS explained on average up to 5.5% variance in ADHD case-control status, when using five different sets of discovery and independent target samples. The SNP-based heritability was calculated as 0.22 (1).

### Target Sample

#### Participants

We used baseline data from the UK Biobank Study (http://www.ukbiobank.ac.uk) (41). A total of 502,655 community-dwelling participants between 37 and 73 years of age were recruited between 2006 and 2010 through the United Kingdom National Health Service patient registers (response rate = 5.47%) and underwent extensive cognitive and physical assessments. We analyzed data on 135,726 individuals (71,874 females) between 40 and 73 years of age (mean ± standard deviation [SD], 56.79 ± 7.96 years) who had available genotyping data after quality control (detailed below). UK Biobank received ethical approval from the Research Ethics Committee (reference 11/NW/0382).

#### Genotyping and Quality Control

A total of 152,729 blood samples were genotyped using either the UK Biobank Lung Exome Variant Evaluation array (*N* = 49,979) or the UK Biobank axiom array (*N* = 102,750). Details on genotyping, quality control, and imputation procedures can be found on the UK Biobank website (http://www.ukbiobank.ac.uk/scientists-3/genetic-data/) and Sudlow *et al.* (37). We further excluded SNPs based on minor allele frequency (<0.01), Hardy-Weinberg equilibrium (*p* < 10^–8^), and missingness (>0.02), and removed participants based on missingness (>0.01), relatedness (>0.088 [*r*∼ = .25]), gender mismatch, and non-Caucasian ancestry. Table 1 shows the sample sizes after quality control for each phenotype. The resulting dataset had 512,536 SNPs and 135,726 samples available for analysis.

**Table 1:** Rates of Diagnoses and Mean Scores on Target Phenotypes

Target Phenotypes	Value	Total, *n*
Continuous Phenotypes, Mean ± SD		
Verbal-numerical reasoning	6.11 **±** 2.11	43,637
Neuroticism	4.11 **±** 3.27	110,213
Alcohol intake frequency	2.89 **±** 1.50	135,586
Body mass index, kg/cm^2^	27.52 **±** 4.84	135,348
Binary Phenotypes, *n* (%)		
Anxiety disorder	2575 (2.14)	120,362
Depressive disorder	8818 (6.96)	126,605
Bipolar disorder	2232 (1.86)	120,019
Schizophrenia	288 (0.24)	118,075
Alcohol dependency	988 (0.83)	118,775
Risk-taking	39,245 (29.00)	135,348
Tobacco use	2911 (2.15)	135,348

Verbal-numerical reasoning score was assessed as the number of correctly answered multiple choice questions (range, 0–13). Neuroticism was assessed as the number of neurotic traits present (range, 0–12). Alcohol intake frequency was scored as follows: 5 = daily or almost daily; 4 = 3 or 4 times a week; 3 = 1 or 2 times a week; 2 = 1 to 2 times a month; and 1 = special occasions only.

#### Phenotypes: BMI

BMI, which is constructed from weight and height (kg/cm^2^), was measured during the initial assessment. BMI values were excluded if data on either height or weight were missing.

#### Phenotypes: General Cognitive Ability

Participants completed a verbal-numerical reasoning test, consisting of 13 multiple choice questions (6 verbal/7 numerical) answered within a 2-minute time period (Supplemental Table S1). The test has shown a satisfactory level of test–retest reliability (*r* = .65) and a high genetic correlation with a general factor of cognitive ability (*r*_g_ = .81, *p* = 6.2 × 10^−18^) 38, 39.

#### Phenotypes: Internalizing Traits and Psychiatric Disorders

Neuroticism was measured using 12 items (Supplemental Table S2) from the Eysenck Personality Inventory Neuroticism Scale–Revised (40). The score of each individual corresponds to the number of neurotic traits present, each coded as a binary variable (1 = yes, 0 = no).

Primary (the most resource-intensive condition) or secondary ICD-10 diagnoses (accessed through hospital records) and self-report measures (reports of having experienced a disorder during an interview with a nurse) were used to identify individuals who had experienced instances of anxiety and depressive disorders, BD, and schizophrenia (ICD-10 codes can be found in Supplemental Table S3). Individuals were indexed as having experienced a psychiatric disorder if they met criteria either through self-report or an ICD-10 diagnosis (any ICD subtype as seen in Supplemental Table S3).

#### Phenotypes: Substance Use and Risk-Taking

Alcohol intake frequency was measured by asking participants “About how often do you drink alcohol?” and was coded on a 5-point scale (Supplemental Table S4). Primary or secondary ICD-10 diagnoses (accessed through hospital records) and self-report measures (reports of having experienced a disorder during an interview with a nurse) were used to identify individuals that had ever experienced alcohol dependency or a mental/behavioral disorder owing to alcohol use (Supplemental Table S3). Information on smoking (ICD-10 code Z72.0) was accessed through hospital records. Risk-taking was measured by asking participants “Would you describe yourself as someone who takes risks?” and was coded as a binary variable (1 = yes, 0 = no).

The control group used for comparisons with the diagnostic groups consisted of individuals that did not have any ICD-10 or self-reported diagnosis of alcohol dependency, anxiety disorder, depressive disorder, BD, or schizophrenia and did not take lithium, antidepressants, or antipsychotics.

We did not investigate participants with ADHD because only 7 individuals had an ICD-10 diagnosis (secondary) for ADHD or were taking stimulant medications (methylphenidate or Ritalin) in our genotyped sample. There were also few participants (*n* < 25) diagnosed with oppositional defiant disorder, conduct disorder, or autism spectrum disorder. The low prevalence rate of ADHD and these other disorders in the UK Biobank is likely related to the older age of the sample (40–73 years of age), as they are most often diagnosed in childhood but were not as commonly recognized when participants were school-aged children.

#### Phenotypes: Control Traits

We also investigated eight “control” phenotypes that we did not expect to be significantly associated with PRS ADHD, in order to confirm that any reported significant results were not caused by the inflation of type I errors. These control traits were height, age, year of initial assessment, menstruation during initial assessment, number of self-reported cancers, hand grip strength, visual acuity, and sex of baby (Supplemental Table S5).

### PRS Analyses

PRSs were computed for each UK Biobank participant using PRSice software (http://www.prsice.info/) (41), with the mega GWAS summary statistics as the discovery dataset. PRSice computes scores by calculating the sum of trait-associated alleles, weighted by the odds ratio generated from a GWAS in an independent sample. An *r*^2^ ≥ .1 (250-kb window) was used for clumping to remove SNPs in linkage disequilibrium. Logistic and linear regression models were used to estimate associations between PRSs and phenotypes in the UK Biobank. PRSs were calculated at a large number of *p* value thresholds for SNP inclusion (“high resolution scoring”) (41) to provide the most predictive PRS. *p* Value thresholds were between *p*_T_ = 0 and *p*_T_ = 0.5 at increments of .001. Results are presented where the most predictive PRS is identified for each phenotype. We set a conservative significance threshold of *p* < 2.1 × 10^−4^ for the main analyses on traits of interest and “control” traits, based on testing the most predictive PRS across 19 phenotypes (see Supplemental Methods).

We controlled for population stratification by conducting analyses with imputed markers and 15 principal components as covariates. We included birthplace, age, and sex as covariates in all analyses, and also batch, in order to control for any genetic differences associated with the batches that samples were analyses in or the genotyping platforms. The *R*^2^ values we report are adjusted from a baseline model including the covariates. In addition, we ran secondary analyses where we explored the effect of sex by including PRS by sex interaction effects. For these analyses, we set a stringent significance threshold of *p* < 4.5 × 10^−4^ (see Supplemental Methods). In the prediction model for height, we added BMI as a covariate because of the significant phenotypic association between BMI and height (*r* = −.0145, *p* = 1.07 × 10^−24^).

## Results

Table 1 summarizes the number of individuals included in analyses for each target phenotype and presents mean values and standard deviations for the continuous phenotypes and the number of “cases” for the binary phenotypes.

### Body Mass Index

**Figure 1:**
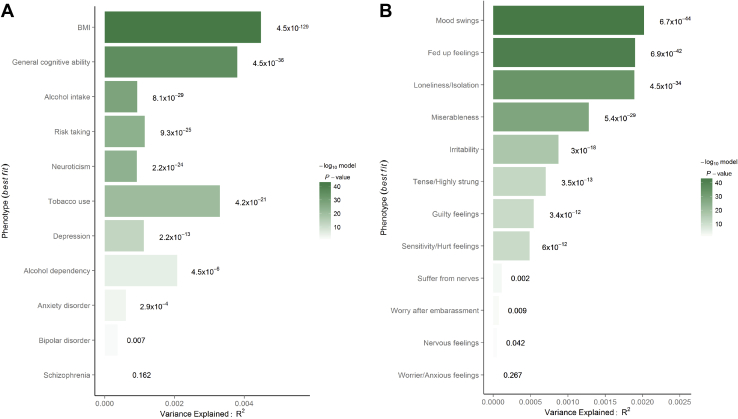
Association between polygenic risk scores for attention-deficit/hyperactivity disorder and **(A)** target phenotypes and **(B)** items on the neuroticism scale. Values displayed next to each bar represent the *p* value for significance for the most predictive models. The significance threshold was set to *p* < 2.1 × 10^−4^. BMI, body mass index.

**Figure 2:**
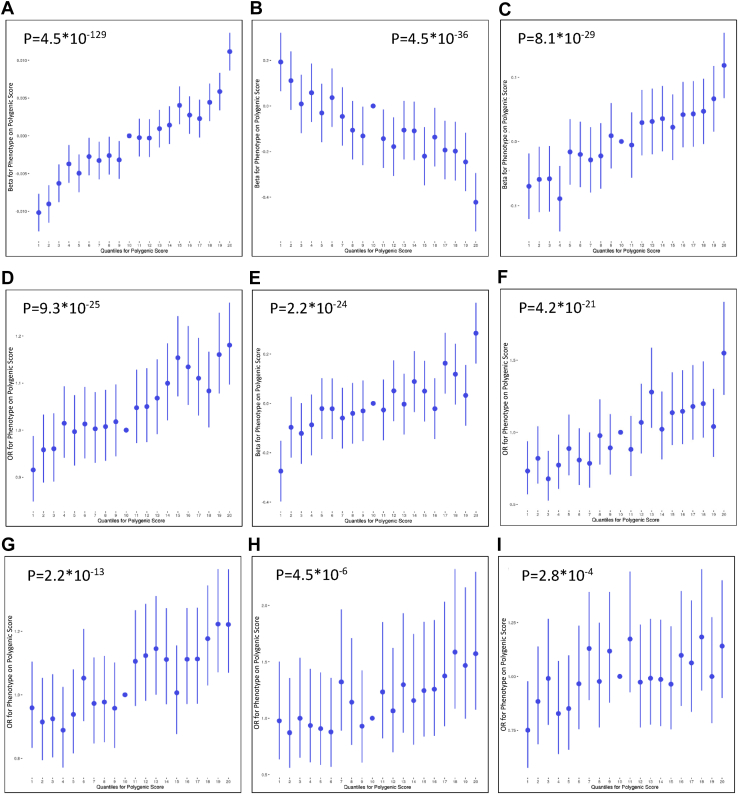
Quantiles of polygenic risk scores plotted against effects on phenotypes. **(A)** Body mass index; **(B)** verbal-numerical reasoning; **(C)** alcohol intake; **(D)** risk-taking; **(E)** neuroticism; **(F)** tobacco use; **(G)** depression; **(H)** alcohol dependency; and **(I)** anxiety disorder. A regression is performed with phenotype as outcome and each 5% quantile separately, whereby the effect size of each quantile is compared to the central quantile as reference, such that each polygenic score in the quantile in question is coded 1 and each polygenic score in the reference quantile is coded 0. In each regression, the covariates used in the main analyses are included. OR, odds ratio.

PRS for ADHD significantly (*p* = 4.5 × 10^−129^) predicted BMI (*R*^2^ = .45%, *p*_T_ = .44) (Figure 1), and the quantile plot demonstrates the positive nature of this relationship as BMI increases with greater polygenic load for ADHD (Figure 2). Mean BMI was significantly higher in males (mean ± SD, 27.95 ± 4.31) than in females (27.14 ± 5.23).

### General Cognitive Ability

PRS for ADHD significantly (*p* = 4.5 × 10^−36^) predicted verbal-numerical reasoning scores (*R*^2^ = .38%, *p*_T_ = .42) (Figure 1), and the quantile plot shows that verbal-numerical reasoning scores decreased with increasing polygenic load for ADHD (Figure 2). Verbal-numerical reasoning test scores were significantly higher in males (6.22 ± 2.18) than in females (6.01 ± 2.05).

### Internalizing Traits and Psychiatric Disorders

PRS for ADHD significantly (*p* = 2.2 × 10^−24^) predicted neuroticism (*R*^2^ = .09%, *p*_T_ = .14) and the quantile plot demonstrates that neuroticism scores increase with higher polygenic load for ADHD (Figure 2). Females showed significantly higher neuroticism levels (4.60 ± 3.26) than males (3.60 ± 3.20). We further investigated the separate 12 neuroticism items (Figure 1). PRS for ADHD significantly and positively predicted mood swings (*R*^2^ = .002%), fed-up feelings (*R*^2^ = .20%), feelings of loneliness and isolation (*R*^2^ = .19%), miserableness (*R*^2^ = .13%), irritability (*R*^2^ = .09%), being tense/highly strung (*R*^2^ = .07%), guilty feelings (*R*^2^ = .05%), and having easily hurt feelings (*R*^2^ = .05%). The PRS did not predict suffering from nerves, often worrying after embarrassment, or being a nervous person or a worrier.

PRS for ADHD also significantly (*p* = 2.2 × 10^−13^) predicted depressive disorder (*R*^2^ = .11%, *p*_T_ = .03) and suggestively (*p* = 2.8 × 10^−4^) predicted anxiety (*R*^2^ = .06%, *p*_T_ = .12) but not BD or schizophrenia (Figure 1). Quantile plots (Figure 2) show that the significant associations were positive. A significantly higher proportion of females than males presented with anxiety (2.6% vs. 1.6%), depression (8.5% vs. 5.2%), and BD (2.3% vs. 1.4%), but the opposite trend was observed for schizophrenia (0.2% vs. 0.3%).

### Substance Use and Risk-Taking

PRS for ADHD significantly (*p* < 2.1 × 10^−4^) predicted risk-taking (*R*^2^ = .12%, *p*_T_ = .29), alcohol intake frequency (*R*^2^ = .09%, *p*_T_ = .23) and dependency (*R*^2^ = .21%, *p*_T_ = .18), and smoking (*R*^2^ = .33%, *p*_T_ = .49). Quantile plots suggest that all of these relationships were positive in nature (Figure 2). A significantly higher proportion of males than females were risk-takers (36.3% vs. 22.3%), alcohol dependent (1.2% vs. 0.4%), and smokers (2.5% vs. 1.8%). Females showed significantly higher alcohol intake frequency (3.14 ± 1.53) than males (2.60 ± 1.42).

We found no significant PRS by sex interaction effects for any of the target phenotypes (Table 2).

**Table 2:** Polygenic Risk Score by Sex Interaction and Main Effects of Sex on Target Phenotypes

Target Phenotype	PRS_Mβ_	PRS_Fβ_	*p*_interaction_	*t/z* Score	Sex_β_	*p*_sex_	*t/z* Score
Body Mass Index	0.07	0.07	.03	−2.23	0.10	1.55 × 10^−725^	35.36
Verbal-Numerical Reasoning	−0.05	−0.07	.12	1.55	0.05	4.82 × 10^−25^	10.34
Alcohol Intake	0.03	0.04	.11	−1.62	−0.18	<2 × 10^−285^	−66.46
Risk-Taking	0.15	0.14	.26	1.13	0.81	<2 × 10^−285^	57.70
Neuroticism	0.03	0.03	.52	−0.64	−0.15	<2 × 10^−285^	−49.19
Tobacco Use	1.10	1.33	.57	−0.56	1.02	5.79 × 10^−14^	7.51
Depressive Disorders	0.45	0.27	.36	0.91	−1.07	2.55 × 10^−116^	−22.93
Alcohol Dependency	1.28	2.93	.36	−0.92	5.45	1.79 × 10^−39^	13.15
Anxiety Disorders	0.37	0.57	.37	−0.89	−1.62	1.69 × 10^−28^	−11.07
Bipolar Disorder	0.21	0.53	.29	−1.06	−1.79	1.73 × 10^−26^	−10.65
Schizophrenia	2.56	0.40	.29	1.06	4.35	.00071	3.39

Significance threshold set at *p* < 4.5 × 10^−4^.

PRS_M/F_, prediction of polygenic risk score on target phenotype for males and females.

### Control Phenotypes

PRS for ADHD significantly (*p* < 2.1 × 10^−4^) and negatively predicted height (*R*^2^ = .03%, *p*_T_ = .08) and age (*R*^2^ = .03%, *p*_T_ = .18), but not any of the remaining six control phenotypes (Table 3). After controlling for educational achievement (detailed in Supplemental Table S6), which has been found to be genetically associated with height (42), the significant association between PRS for ADHD and height was no longer significant (*R*^2^ = .005%, *p* = .0001); however, the association between PRS and age remained and was significant in both males (*R*^2^ = .021%, *p* = 3 × 10^−5^) and females (*R*^2^ = .029%, *p* = 8 × 10^−6^). When we reran all the main analyses controlling for educational achievement and BMI, which were the two additional covariates in the PRS–height model, the overall pattern of results remained the same, although effect sizes decreased for most traits (Supplemental Table S7).

**Table 3:** Prediction of Polygenic Risk Score for Attention-Deficit/Hyperactivity Disorder on Target and Control Phenotypes

Target or Control Phenotype	*p*	*p*_T_	*R*^2^ (%)	SNPs, *n*
Body Mass Index	4.5 × 10^−129^	.440	.448	69,995
Verbal-Numerical Reasoning	4.5 × 10^−36^	.418	.379	67,558
Alcohol Intake Frequency	8.1 × 10^−29^	.231	.093	44,307
Risk-Taking	9.3 × 10^−25^	.291	.115	52,388
Neuroticism	2.2 × 10^−24^	.139	.092	30,306
Tobacco Use	4.2 × 10^−21^	.485	.333	74,809
Height	8.7 × 10^−20^	.081	.030	20,147
Depressive Disorder	2.2 × 10^−13^	.033	.112	10,158
Age, Years	5.8 × 10^−9^	.177	.026	36,443
Alcohol Dependency	4.5 × 10^−6^	.175	.208	36,101
Anxiety Disorder	2.8 × 10^−4^	.116	.062	26,355
Visual Acuity	.005	.001	.029	792
Bipolar Disorder	.007	.117	.037	26,551
Hand Grip Strength	.024	.494	.002	75,689
Menstruation at Assessment	.115	.051	.025	14,128
No. of Cancers	.127	.131	.002	28,929
Schizophrenia	.162	.257	.053	47,870
Year of Assessment	.159	.036	.001	10,871
Sex of Child	.234	.010	.062	4085

Significance threshold set at *p* < 2.1 × 10^−4^.

SNP, single nucleotide polymorphism.

Table 3 and Supplemental Figures S1 to S11 provide more detailed information and plots for the PRS prediction models.

## Discussion

Using PRSs derived from the recently published mega GWAS (1), we found that polygenic risk for clinically diagnosed ADHD predicts higher BMI, neuroticism, risk-taking, tobacco and alcohol use, and anxiety and depressive disorders, and lower general cognitive ability in an adult population sample. These are the first reports of significant genetic associations between ADHD and neuroticism traits, risk-taking, and alcohol use based on genome-wide data. The remaining associations are consistent with a relatively limited literature of studies demonstrating pleiotropy of the genetic variants underlying ADHD. No sex-specific effects were observed in relation to the association between PRS for ADHD and co-occurring features.

Individuals with many risk alleles for ADHD were more likely to have higher BMI than those with few risk alleles. There is limited research investigating why ADHD and high BMI often co-occur, but our findings, together with recent findings using LDSR 1, 35, suggest that they have an overlapping genetic basis. Further research is needed to identify genetic pathways and neurobiological mechanisms relating to this genetic overlap, which could prove vital for improving prevention and treatment interventions for individuals with ADHD who are at risk of obesity. One possibility is that dopaminergic pathways and pathways implicated in eating patterns (e.g., binge- and emotional-eating), sleeping patterns, and sedentary behavior explain the association between ADHD and BMI, which would be in line with initial evidence 42, 43, 44, 45, 46. The common mechanisms underlying both ADHD and BMI could either reflect biological pleiotropy, where similar mechanisms influence both traits, or mediated pleiotropy, where certain mechanisms influences one of the traits, which in turn influences the other.

Polygenic risk for ADHD was significantly associated with lower cognitive ability, which is in line with previous twin and molecular genetic studies 2, 29, 30, 35. The association between ADHD and general cognitive ability is thought to be mainly driven by ADHD symptoms that influence IQ, at least in adolescence (47). It may therefore be possible that there are common biological mechanisms underlying both ADHD and IQ, but perhaps also certain biological mechanisms underlie ADHD, which in turn influences IQ, possibly through poor educational achievement owing to difficulties concentrating in school 47, 48.

Polygenic risk for ADHD significantly and positively predicted neuroticism, including individual items such as mood swings and irritability. Two recent studies failed to find any genetic correlation between ADHD and neuroticism using LDSR 35, 49. The discrepancy in findings may be due to the previous studies having smaller sample sizes or the use of LDSR rather than polygenic scoring, potentially resulting in insufficient statistical power to detect effects.

PRSs for ADHD also predicted depression, and anxiety at a suggestive level, which is in line with findings from twin and genome-wide studies 9, 31, 32, 33, 35. The ADHD PRSs did not predict BD or schizophrenia; however, these results should be considered with caution because previous family-based and genome-wide studies using other statistical methods have reported significant genetic associations between these disorders 34, 35. The discrepancy in findings may be related to the older age of our sample, the use of a population cohort rather than clear case-control groups, or insufficient power to detect effects, in particular for schizophrenia (288 cases) based on power calculations using Avengeme R package (power for analyses: BD = 0.99, schizophrenia = 0.22). Further polygenic studies are needed to investigate the association of ADHD with BD and schizophrenia across different study populations to clarify the true etiological relationship between the disorders.

Individuals with many risk alleles for ADHD were more likely to display alcohol dependency, have higher alcohol intake frequency, and be smokers and risk-takers compared with those with few risk alleles. Previous genome-wide studies reported significant genetic associations between ADHD and smoking 35, 50, 51 but not between ADHD and alcohol use (52), and no studies to our knowledge have investigated the genetic association between ADHD and risk-taking. The shared genetic risk between ADHD and these risk-taking and health-related outcomes may be explained by common neurobiological mechanisms involved in self-regulation and inhibitory control. Further research targeting relevant genes and pathways is needed to test such hypotheses.

Overall, our findings lend support for the continuous nature of ADHD across the entire population. We find that common risk alleles that contribute to clinically diagnosed ADHD also influence common traits and disorders in the general population, across ages, which suggests that ADHD symptoms represent continuous traits and that similar genetic influences may be present in younger and older individuals. This fits well with the current understanding of ADHD based on evidence from behavioral, family-based, and genetic studies 53, 54, 55.

To investigate if our significant results could be the result of type I errors, we examined if PRSs for ADHD significantly predicted several “control” phenotypes that were not expected to be associated with polygenic risk for ADHD. Out of the eight “control” traits, only age was significantly predicted by ADHD PRS. It is possible that this association is caused by some real effect, such as genetic influences on ADHD being stronger during certain developmental periods, for example in childhood, when the prevalence of ADHD is the highest. Twin studies suggest that the heritability of childhood ADHD is stronger than in adult ADHD, but this may also be due to rater effects (56). Hypothetically, this would then have been captured in the discovery GWAS, where genetic effect sizes in children would be larger than in adults and in turn lead to PRS associations with younger age in the UK Biobank. However, we cannot rule out the possibility that the “age” result reflects a false positive or is related to the overlap between UK Biobank participants and those of the Psychiatric Genomics Consortium/iPSYCH ADHD GWAS, which may cause slight inflation in results. It is reassuring, however, that seven of eight control traits showed nonsignificant results and that the relative strength of the significant results are in line with other preliminary genetic findings.

An advantage of using a large dataset and the PRS approach is that we could directly investigate sex differences in the relationship between PRSs and the target phenotypes. A recent study based on the ADHD mega GWAS data found a strong genetic correlation for ADHD across sex and no difference in polygenic load across sex (36), and we extend these findings to show that the polygenic influences underlying the relationship between ADHD and co-occurring features are similar across men and women.

### Limitations and Future Directions

One should interpret our findings in light of the study limitations. Our study participants were between 40 and 73 years of age, had a lower prevalence of mental health disorders, and were recruited within the United Kingdom. It would be informative to investigate the generalizability of our findings by replicating the analyses using participants of different age groups and from different populations. Selection bias of the sample could also have influenced the associations we report (57); however, we controlled for several important measures, including age and birthplace, to minimize the chance for bias. In addition, several of the significant genetic associations that we identified confirm previous statistical genetic findings (35), offering some validation of our results. PRSs explain only a tiny fraction of the variance in the target phenotypes, and obtaining a complete picture of the etiological overlap between ADHD and co-occurring features will require larger sample sizes and inclusion of other genetic factors, such as copy number and rare variants.

In conclusion, higher polygenic load for clinical ADHD was associated with higher BMI, neurotic and risk-taking behavior, anxiety and depressive disorders and substance use, and lower general cognitive ability in the general population. These findings suggest that the co-occurrence of several traits and disorders with ADHD are partly explained by the same common genetic factors. Further investigations are needed to determine the specific neurobiological mechanisms associated with the shared genetic etiology between ADHD and co-occurring features.
