# Supplementary material for: Association of Polygenic Risk for Attention-Deficit/Hyperactivity Disorder With Co-occurring Traits and Disorders
Source: Biol Psychiatry Cogn Neurosci Neuroimaging. 2018 Jul;3(7):635–43. doi: 10.1016/j.bpsc.2017.11.013 (PMC6278881; doi:10.1016/j.bpsc.2017.11.013)
Supplement: Supplemental Material [file mmc1.pdf]

Association of Polygenic Risk for Attention-Deficit/Hyperactivity Disorder With Co-Occurring Traits and Disorders

Supplementary Information

Supplemental Methods. Significance thresholds

Table S1. List of items in the Verbal-Numerical Reasoning test

Table S2. List of items in the Eysenck Personality Inventory Neuroticism scale – Revised (EPIN-R)

Table S3. List of ICD-10 codes for psychiatric diagnoses used as target phenotypes

Table S4. List of responses for alcohol intake frequency measure

Table S5. Detailed summary of control phenotypes

Table S6. Detailed summary of educational achievement

Table S7. Predictive accuracy of PRS on the target phenotypes after controlling for BMI and educational achievement

Figure S1. Plot for alcohol dependency

Figure S2. Plot for alcohol intake frequency

Figure S3. Plot for anxiety

Figure S4. Plot for bipolar disorder

Figure S5. Plot for BMI

Figure S6. Plot for depressive disorder

Figure S7. Plot for verbal-numerical reasoning

Figure S8. Plot for neuroticism

Figure S9. Plot for risk-taking

Figure S10. Plot for schizophrenia

Figure S11. Plot for tobacco use

Supplemental Methods

Significance thresholds

For our primary PRS analyses, we selected a conservative significance threshold to control for multiple testing by applying a Bonferroni correction. Euesden et al.(1) recommend using a significance threshold of at least P=0.004 in order to control for the high-resolution scoring approach of selecting the most predictive PRS. As we tested the most predictive PRS across each of the 11 target phenotypes, we divided the P-value by the number of tests performed (P=0.004/19), which resulted in a significance threshold of $P=2.1x10^{-4}$.

For our secondary analyses, where we tested the main effects of sex and PRS*sex interaction effects, we selected a conservative significance threshold of P=0.01 as we did not have strong a priori hypotheses of the effects. We applied Bonferroni correction in order to control for the 22 secondary analyses we ran (P=0.01/22), which resulted in a significance threshold of $P=4.5x10^{-4}$.

1. Euesden J, Lewis CM, O'Reilly PF. PRSice: Polygenic Risk Score software. *Bioinformatics*. 2015;31(9): 1466-1468.

Table S1: List of items in the Verbal-Numerical Reasoning test

Question	Possible answers
Add the following numbers together: 1 2 3 4 5 – is the answer?	13/14/15/16/17/Do not know/Prefer not to answer
Which number is the largest?	642/308/987/714/253/Do not know/Prefer not to answer
Bud is to flower as child is to?	Grow/Develop/Improve/Adult/Old/Do not know/Prefer not to answer
11 12 13 14 15 16 17 18 Divide the sixth number to the right of twelve by three. Is the answer?	5/6/7/8/Do not know/Prefer not to answer
If Truda’s mother’s brother is Tim’s sister’s father, what relation is Truda to Tim?	Aunt/Sister/Niece/Cousin/No relation/Do not know/Prefer not to answer
If sixty is more than half of seventy-five, multiply twenty-three by three. If not subtract 15 from eighty-five. Is the answer?	68/69/70/71/72/Do not know/Prefer not to answer
Stop means the same as?	Pause/Close/Cease/Break/Rest/Do not know/Prefer not to answer
If David is twenty-one and Owen is nineteen and Daniel is nine years younger than David, what is half their combined age?	25/26/27/28/29/Do not know/Prefer not to answer
Age is to years as height is to?	Long/Deep/Top/Metres/Tall/Do not know/Prefer not to answer
150...137...125...114...104... What comes next?	96/95/94/93/92/Do not know/Prefer not to answer
Relaxed means the opposite of?	Calm/Anxious/Cool/Worried/Tense/Do not know/Prefer not to answer
100...99...95...86...70...What comes next?	50/49/48/47/46/45/Do not know/Prefer not to answer
If some flinks are plinks and some plinks are stinks then some flinks are definitely stinks?	False/True/Neither true nor false/Not sure/Do not know/Prefer not to answer

Table S2: List of items in the Eysenck Personality Inventory Neuroticism scale – Revised (EPIN-R)

Question	Possible answers
Does your mood often go up and down?	Yes/No/Do not know/Prefer not to answer
Do you ever feel ‘just miserable’ for no reason?	
Are you an irritable person?	
Are your feelings easily hurt?	
Do you often feel ‘fed-up’?	
Would you call yourself a nervous person?	
Are you a worrier?	
Would you call yourself tense or ‘highly strung’?	
Do you worry too long after an embarrassing experience?	
Do you suffer from ‘nerves’?	
Do you often feel lonely?	
Are you often troubled by feelings of guilt?	

Note: If individuals responded ‘Do not know’ or ‘Prefer not to answer’, they were excluded from analyses.

Table S3: List of ICD-10 codes for psychiatric diagnoses used as target phenotypes

**Psychiatric diagnoses**	**ICD-10 codes**
Anxiety and stress-related disorders	
Phobic anxiety disorders	F40
Other anxiety disorders	F41
Obsessive-compulsive disorder	F42
Reaction to severe stress, and adjustment disorders (includes post-traumatic stress disorder)	F43
Depressive disorders	
Depressive episode	F32
Recurrent depressive disorder	F33
Bipolar affective disorder	F31
Schizophrenia, schizotypal and delusional disorders	
Schizophrenia	F20
Schizotypal disorder	F21
Persistent delusional disorders	F22
Acute and transient psychotic disorders	F23
Induced delusional disorder	F24
Schizoaffective disorders	F25
Other nonorganic psychotic disorders	F28
Unspecified nonorganic psychosis	F29
Mental and behavioural disorders due to use of alcohol	
Acute intoxication	F10.0
Harmful use	F10.1
Dependence syndrome	F10.2
Withdrawal state	F10.3
Withdrawal state with delirium	F10.4
Psychotic disorder	F10.5
Amnesic syndrome	F10.6
Residual and late-onset psychotic disorder	F10.7
Other mental and behavioural disorders	F10.8
Unspecified mental and behavioural disorder	F10.9

Table S4: List of responses for alcohol intake frequency measure

Question	Possible answers
About how often do you drink alcohol?	1=Daily or almost daily<br>2=Three or four times a week<br>3=Once or twice a week<br>4=One to three times a month<br>5=Special occasions only

Note: Answers were reverse-coded and individuals who responded ‘Do not know’ or ‘Prefer not to answer’, were excluded from analyses.

Table S5: Detailed summary of control phenotypes

Phenotype	Description	Covering N genotyped participants	Mean (SD)/ N Cases (%)
Height (cm)	Standing height measured in centimeters during initial assessment	135,495	168.70 (9.21)
Age (years)	Age when attended initial assessment, derived from date of birth and date of attending assessment centre and truncated to whole year	135,726	56.79 (7.96)
Year of initial assessment	Year when participants came in for initial assessment	135,726	2008.57 (0.88)
Number of self-reported cancers	Number of self-reported cancers recorded using a touch-screen self-completed questionnaire followed by an interview at initial assessment	135,146	0.09 (0.31)
Hand grip strength	Hand grip strength of left hand measured using a Jamar J00105 hydraulic hand dynamometer. This measures grip force isometrically in kilograms.	135,163	29.89 (11.35)
Visual acuity	Visual acuity of left eye measured as the smallest size letters that can be reliably identified at a specified distance. The UK Biobank system is based on a traditional LogMar chart with data captured by Direct Entry to Vox.	29,326	0.02 (0.21)
Menstruating during initial assessment	Question: “Are you menstruating today? (We are asking this as it may affect the urine sample that you have been asked to provide)”. Possible answers: Yes, No, Do not know, Prefer not to answer.	18,829	2,781 (15%)
Sex of baby	Sex of baby refers to the sex of the participants’ child as recorded across all episodes in hospitals. Possible codes: 0=Not known, 1=Male, 2=Female, 3= Indeterminate, 9= Not specified.	3,645	2,818 female (77%)

Table S6: Detailed summary of educational achievement

Description of education variable	Items
Education was based on self-report of highest qualification achieved.	1=College or University degree<br>2=A levels/AS levels or equivalent<br>3=O levels/GCSEs or equivalent<br>4=CSEs or equivalent<br>5=NVQ or HND or HNC or equivalent<br>6=Other professional qualifications e.g.: nursing, teaching<br>-7=None of the above<br>-3=Prefer not to answer

Note: Participants that replied ‘None of the above’ or ‘Prefer not to answer’ were excluded from analyses.

Table S7: Predictive accuracy of PRS on the target phenotypes after controlling for BMI and educational achievement

	**P**	**P_T**	**R^2 (%)**
BMI	2.7*10-99	0.121	0.342
Verbal-numerical reasoning	2.9*10-9	0.418	0.070
Alcohol intake frequency	2.7*10-5	0.231	0.013
Risk taking	1.2*10-25	0.291	0.120
Neuroticism	3.5*10-12	0.139	0.045
Tobacco use	1.6*10-13	0.488	0.230
Depressive disorder	1.7*10-8	0.033	0.067
Alcohol dependency	2.6*10-5	0.175	0.177
Anxiety disorder	0.005	0.116	0.037
Bipolar disorder	0.041	0.117	0.022
Schizophrenia	0.280	0.263	0.032

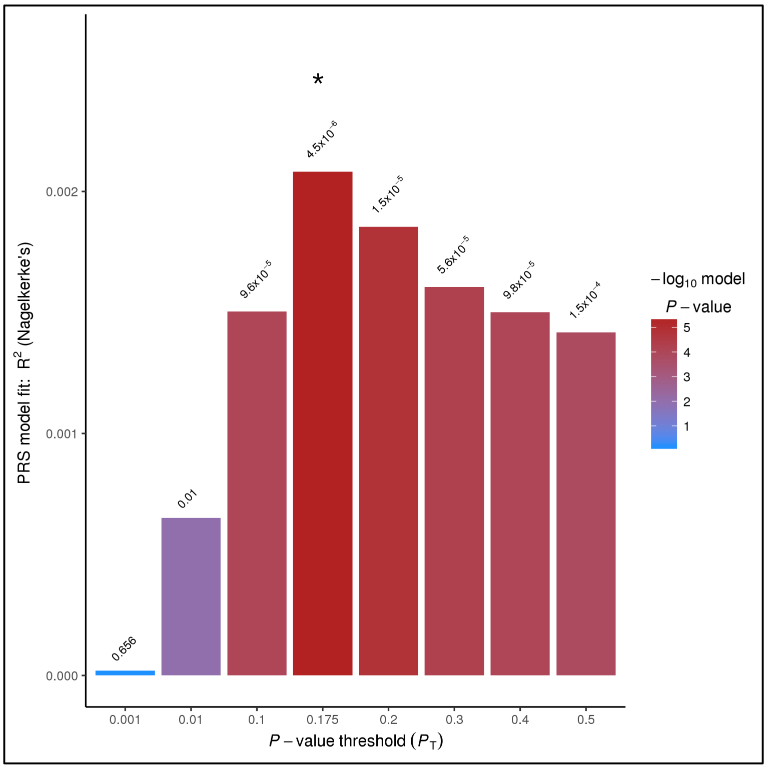

Figure S1: Plot for alcohol dependency

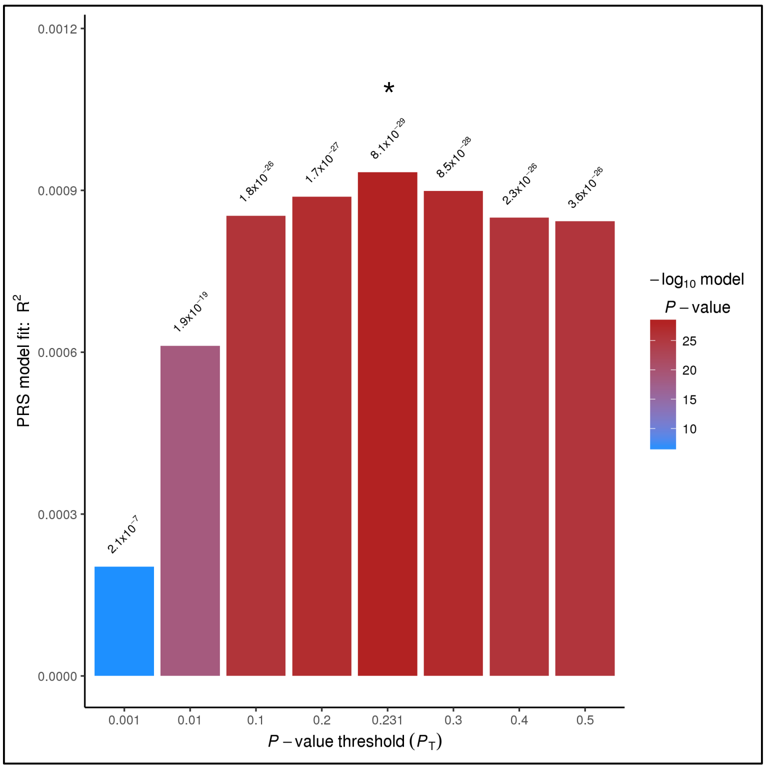

Figure S2: Plot for alcohol intake frequency

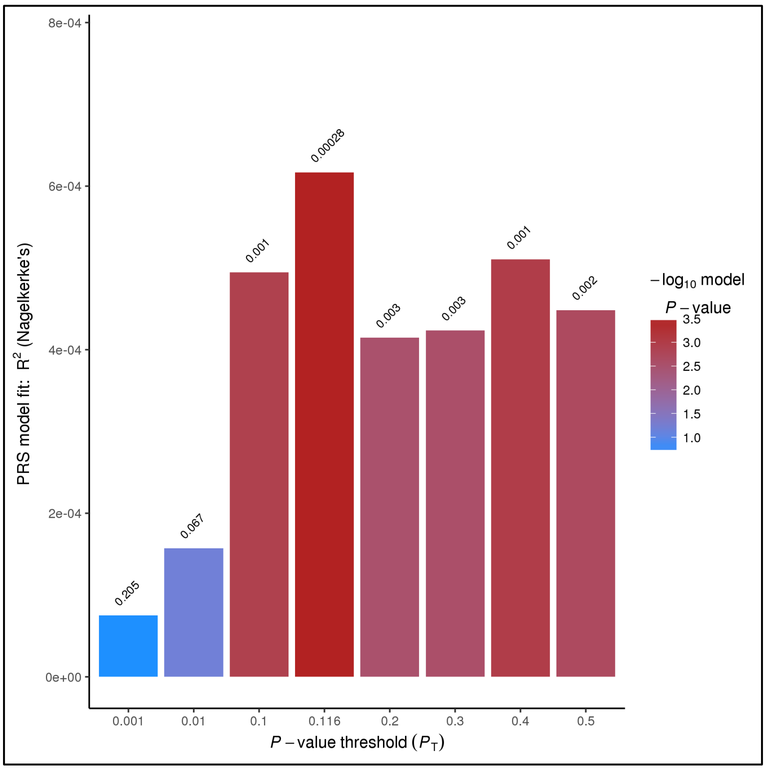

Figure S3: Plot for anxiety

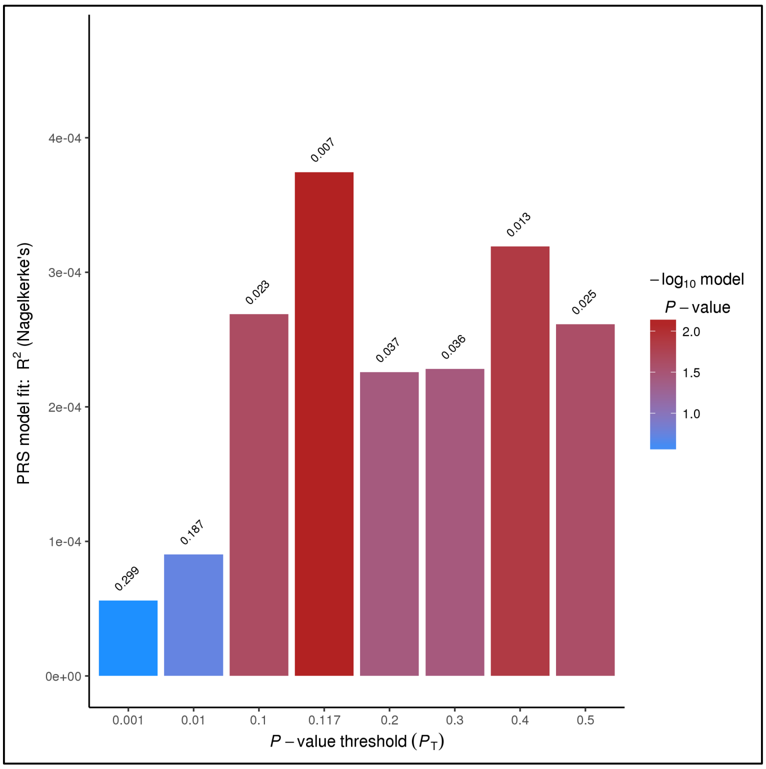

Figure S4: Plot for bipolar disorder

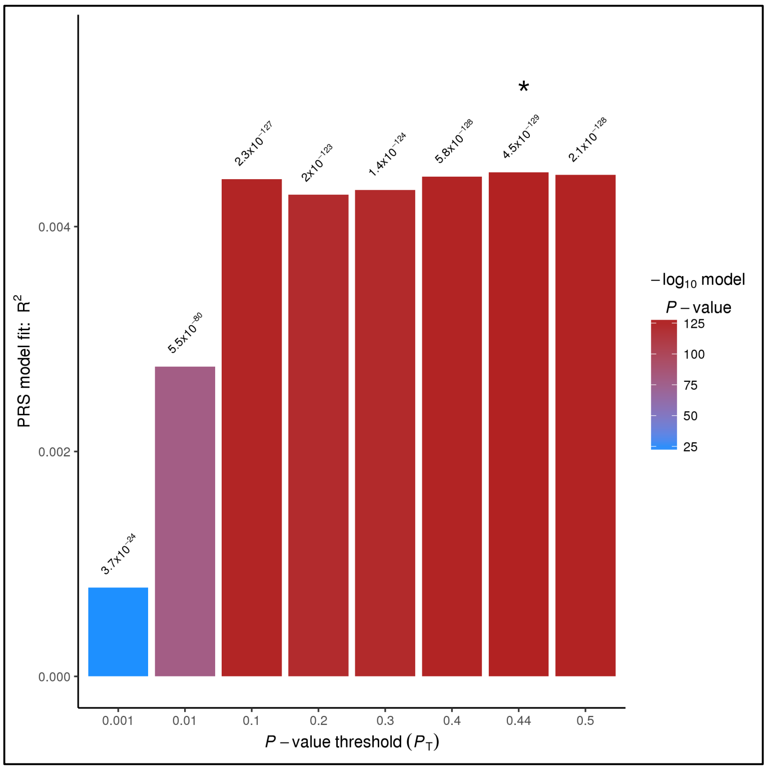

Figure S5: Plot for BMI

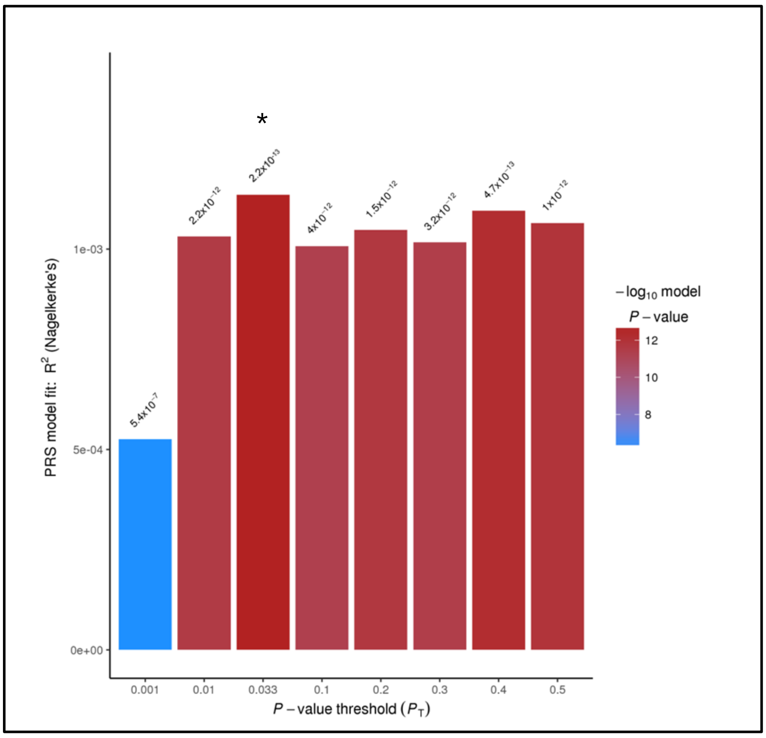

Figure S6: Plot for depressive disorder

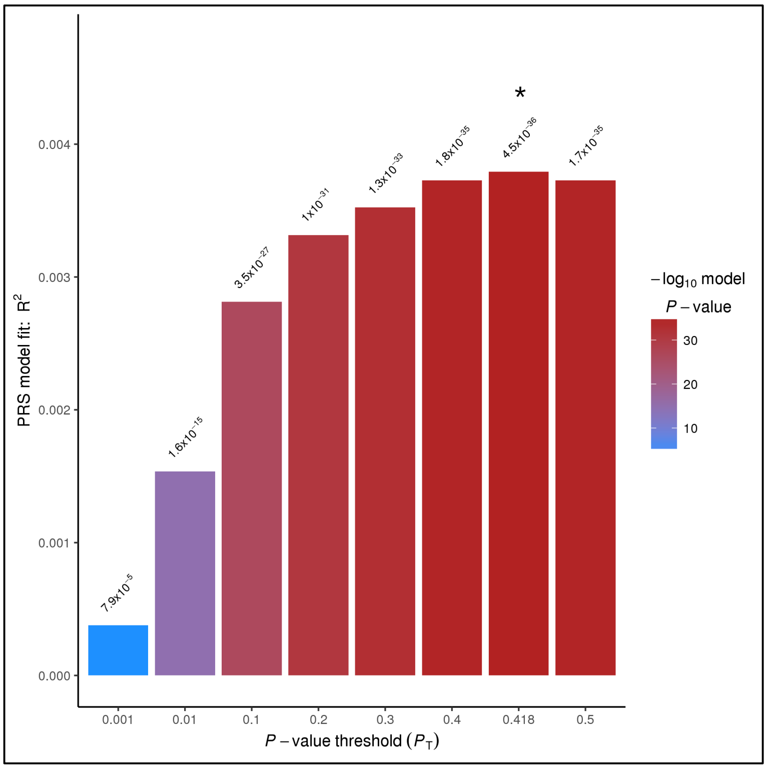

Figure S7: Plot for verbal-numerical reasoning

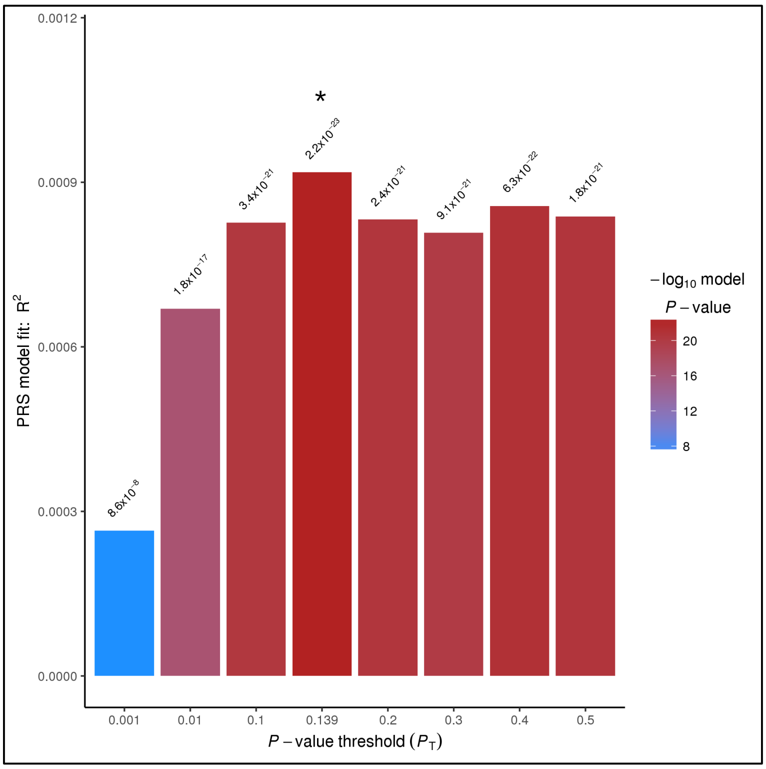

Figure S8: Plot for neuroticism

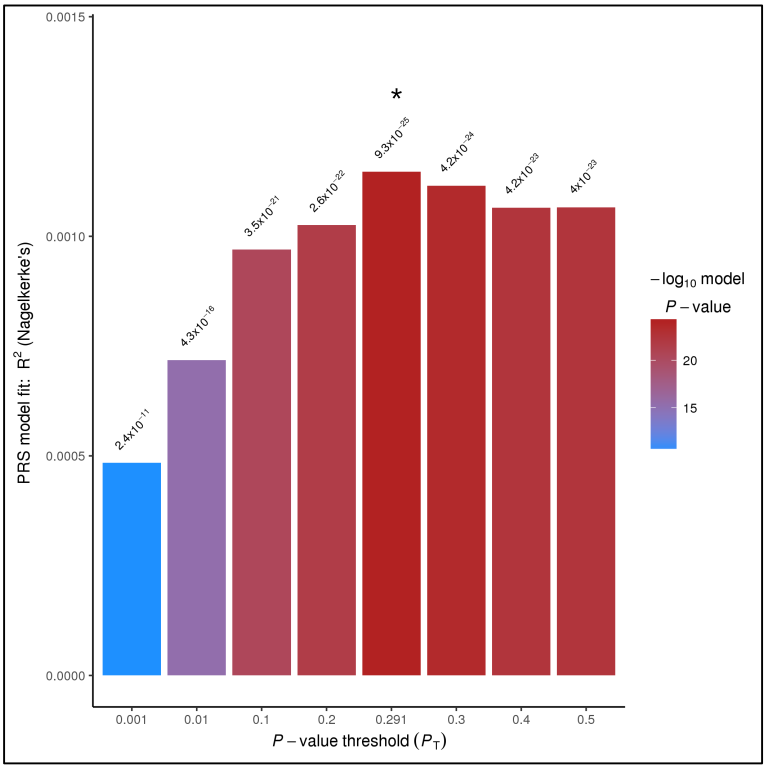

Figure S9: Plot for risk taking

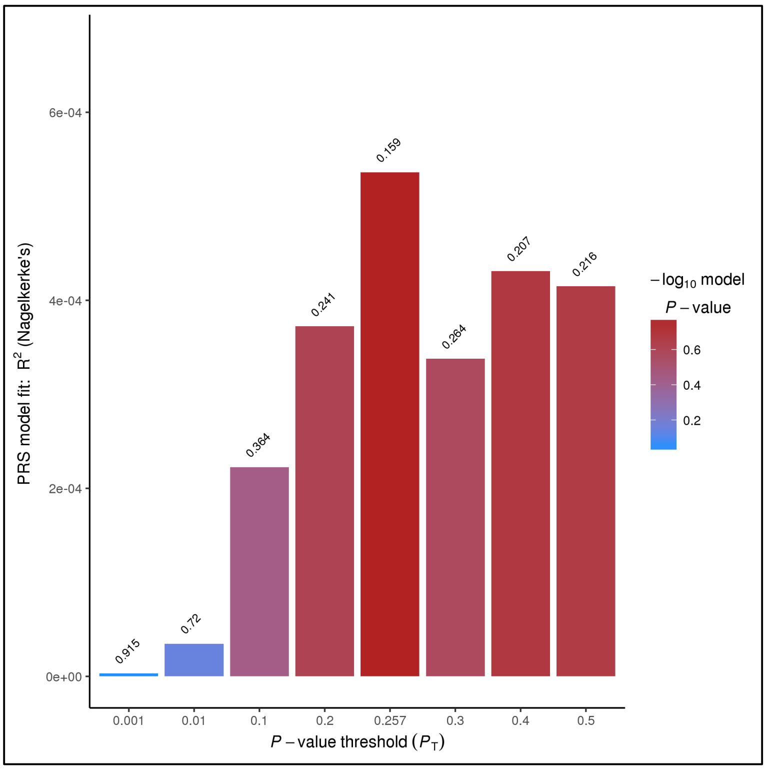

Figure S10: Plot for schizophrenia

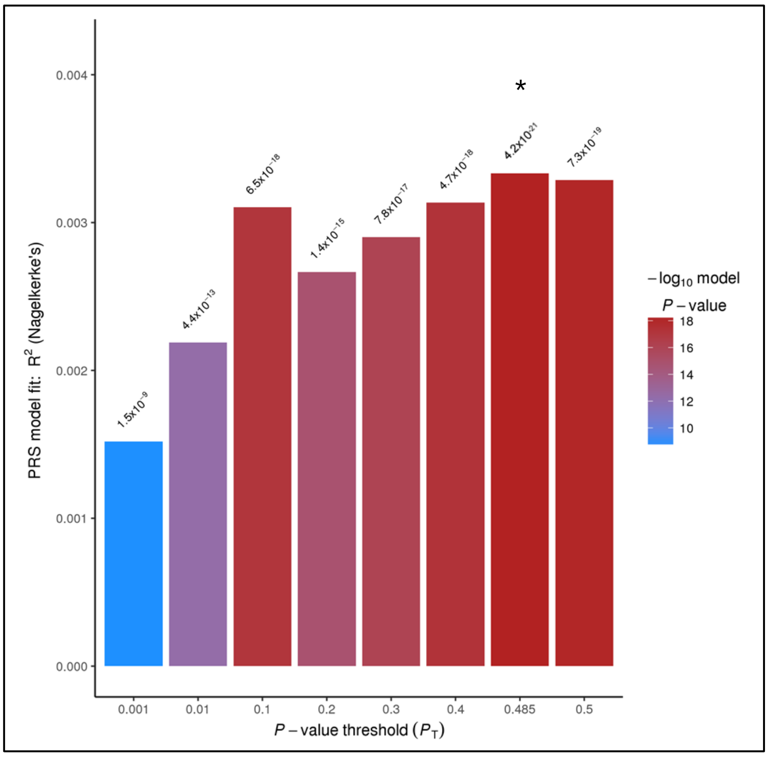

Figure S11: Plot for tobacco use

Note: P-value threshold represents the P-value at the cut-off for inclusion of single-nucleotide polymorphisms in the polygenic risk score. Values on top of the bars represent P-values for the regression models.

* p-value below significance threshold.
